# Chromosome Instability; Implications in Cancer Development, Progression, and Clinical Outcomes

**DOI:** 10.3390/cancers12040824

**Published:** 2020-03-29

**Authors:** Raghvendra Vishwakarma, Kirk J. McManus

**Affiliations:** 1 Research Institute in Oncology & Hematology, CancerCare Manitoba, Winnipeg, MB R3E 0V9, Canada; raghvendra2375@gmail.com; 2 Department of Biochemistry & Medical Genetics, University of Manitoba, Winnipeg, MB R3E 0J9, Canada

**Keywords:** chromosome instability, genome instability, aneuploidy, cancer, tumor heterogeneity, prognosis, metastasis, clinical outcome, therapeutic targeting, chemoresistance

## Abstract

Chromosome instability (CIN) refers to an ongoing rate of chromosomal changes and is a driver of genetic, cell-to-cell heterogeneity. It is an aberrant phenotype that is intimately associated with cancer development and progression. The presence, extent, and level of CIN has tremendous implications for the clinical management and outcomes of those living with cancer. Despite its relevance in cancer, there is still extensive misuse of the term CIN, and this has adversely impacted our ability to identify and characterize the molecular determinants of CIN. Though several decades of genetic research have provided insight into CIN, the molecular determinants remain largely unknown, which severely limits its clinical potential. In this review, we provide a definition of CIN, describe the two main types, and discuss how it differs from aneuploidy. We subsequently detail its impact on cancer development and progression, and describe how it influences metastatic potential with reference to cancer prognosis and outcomes. Finally, we end with a discussion of how CIN induces genetic heterogeneity to influence the use and efficacy of several precision medicine strategies, including patient and risk stratification, as well as its impact on the acquisition of drug resistance and disease recurrence.

## 1. Introduction

For over a century, cancer researchers and oncologists have sought to identify and characterize the molecular determinants (e.g., defective genes, proteins, and cellular pathways) driving cancer development and progression to influence health outcomes. Since the original identification of the Philadelphia chromosome in 1959 [1] and the subsequent discovery of the *BCR:ABL* fusion [2] in chronic myelogenous leukemia, significant efforts have been aimed at identifying the causative genes driving cancer pathogenesis. Traditionally, this quest was fueled by the singular goal of identifying the genetic aberrations exhibiting similar causal relationships in other cancer types; however, it became readily apparent that this cause (*BCR*:*ABL*) and effect (chronic myelogenous leukemia) relationship was more an exception than the rule. Indeed, recent efforts, including both small and large scale cancer genome sequencing projects, have determined that the majority of genetic alterations in a given cancer type are not shared among patients [3,4]. In fact, many cancers exhibit a striking degree of genetic heterogeneity encompassing both small (e.g., single nucleotide alterations, small insertions, or deletions) and large (e.g., gene amplifications/deletions, complex chromosome alterations, and whole chromosome gains/losses) scale alterations. This degree of genetic complexity challenged the classically held belief that genetic alterations of only a small subset of genes were required to drive cancer pathogenesis [5]. Accordingly, there is now a renewed focus on identifying and characterizing the molecular determinants of genome instability, as well as determining their impact on disease development, progression, drug resistance, and clinical outcomes [6,7].

## 2. Genome and Chromosome Instability; Definition and Types of CIN

Genome instability is an enabling hallmark of cancer [8] that facilitates the acquisition of genetic alterations that are instrumental to the development and progression of virtually all cancer types [9]. In general, genome instability describes a succession of genetic alterations within a cell that can include changes in the primary nucleic acid sequence (mutations, insertions, or deletions), chromosome rearrangements (translocations), and aneuploidy (single, multiple, or entire sets of chromosomes are gained or lost). Thus, the term ‘genome instability’ globally defines a spectrum of genetic aberrations ranging from subtle nucleotide changes to extreme genomic changes, including numerical and structural chromosome defects.

While numerous aberrant pathways underlie genome instability (e.g., microsatellite instability (MSI) and CpG island methylator phenotype (CIMP)), chromosome instability (CIN)—or ongoing changes in chromosome complements—is arguably one of the most prevalent but least understood mechanisms. CIN is defined as an increase in the ‘rate’ at which whole chromosomes or large chromosome fragments are gained or lost, and is a driver of cell-to-cell heterogeneity [10]. Thus, the accurate assessment of CIN mandates the use of either: (1) quantitative approaches that are capable of assessing chromosome changes within a continuously growing clonal-derived population over time (temporal) such that a ‘rate’ of change can be calculated or (2) single cell approaches that are capable of quantifying cell-to-cell heterogeneity in genetic and/or chromosome changes within a population of cells at given time point (endpoint) [11]. Furthermore, there are critical distinctions within the CIN phenotype itself, as it can be further subdivided into two main categories: (1) numerical CIN (N-CIN), which involves gains or losses of whole chromosomes, and (2) structural CIN (S-CIN), which describes changes involving large chromosome fragments that can include rearrangements, translocations, amplifications, or deletions. Distinguishing between N- and S-CIN can provide important insights into the etiological origins of CIN, as mitotic defects such as chromosome congression, segregation, or cytokinesis errors typically lead to N-CIN, while genotoxic stress, telomere dysfunction, and defective DNA double strand break repair are most often associated with S-CIN [12]. However, it should be noted that N- and S-CIN are not mutually exclusive, and that both can co-exist within a given cell or tumor (reviewed in [11]).

### 2.1. Critical Distinctions Between Aneuploidy and CIN

It is important to note that aneuploidy is not synonymous with CIN, as aneuploidy describes a ‘state’ of abnormal chromosome numbers, whereas CIN defines an ongoing ‘rate’ of change in chromosome complements. This distinction is critical, as there are a number of genetic syndromes in which all cells in the human body are aneuploid but do not inherently exhibit CIN. For example, cells from individuals with Patau syndrome (trisomy 13), Edward syndrome (trisomy 18) and Down syndrome (trisomy 21), or those from Klinefelter and Jacob syndromes (harbor an extra sex chromosomes), all contain 47 chromosomes and therefore exhibit an aneuploid ‘state,’ rather than an ongoing ‘rate’ of chromosome changes. Interestingly, some of these syndromes are associated with an increased risk of developing cancer [13], raising the possibility that aneuploidy may promote CIN in certain individuals or tissues. Accordingly, it remains to be determined whether congenital aneuploidies are associated with CIN, as evidence exists to suggest that some congenital aneuploidies may promote CIN, at least in amniocytes [14,15]. Nevertheless, the fact that none of these congenital aneuploidies confer a 100% risk indicates that the aneuploid ‘state’ by itself is not sufficient to induce cancer. Apart from the genetic syndromes listed above, aneuploidy may also arise as a stochastic response to cellular stresses that interfere with normal chromosome transmission to daughter cells and is often associated with decreased cell fitness [16]. In this context, the presence of aneuploidy is often associated with accelerated senescence, proliferation defects, apoptosis, and cell death [17,18], which contrast with the phenotypes typically associated with CIN and oncogenesis.

A fundamental issue in distinguishing between aneuploidy and CIN is that cytogeneticists typically ascribe a single modal karyotype (e.g., population average) to a given clinical sample or experimental condition, which effectively negates the cell-to-cell heterogeneity indicative of CIN. This is particularly relevant in the context of numerous genetic studies showing that aneuploid cells frequently exhibit phenotypic defects in many of the same pathways that cause CIN, including increases in micronucleus formation, chromosome missegregation events, cytokinetic defects, and anaphase bridges [19]. Thus, it is critical that researchers employ more accurate tools and approaches to clearly distinguish between aneuploidy and/or CIN (N- or S-CIN) within their studies (reviewed in [11]).

### 2.2. Fundamental Concepts in Assessing CIN: Benefits and Limitations

As indicated above, there are two general strategies to assess CIN: (1) quantifying ongoing changes in chromosome complements of a cell and its progeny over time (temporal approach) or, (2) quantifying the cell-to-cell heterogeneity existing within a given population at a single time point (endpoint approach). In general, temporal approaches for either experimental or clinical purposes require the repeated sampling and analysis of cellular populations over time. This can be more easily achieved in laboratory settings through the use of continual, long-term culturing techniques, but this repeated sampling becomes more complicated in clinical settings due to the complexities associated with different cancer types. For example, tumor cells can be readily isolated and assessed from hematological malignancies through repeat phlebotomies, while solid tumors could conceivably be assessed through repeat biopsies, although repeat biopsies are associated with an increased risk for metastatic spread [20,21] (see Section 6.1). Alternatively, minimally invasive approaches including the isolation and analysis of circulating tumor cells (CTCs) or tumor cells isolated from serial ascites samples (fluid accumulation within the peritoneal cavity containing tumor cells) could be employed; however, ascites is only associated with certain tumor types and in only a small subset of individuals. In fact, Penner-Goeke and colleagues [22] recently identified unique temporal dynamics for CIN in drug-resistant and recurrent high-grade serous ovarian cancer. Furthermore, while the temporal approach allows for the calculation of an exact ‘rate’ of change, it is limited in that CIN is a highly heterogeneous phenotype that is not expected to be associated with a single ‘rate’—rather, a spectrum of rates within a given population. Consequently, the temporal approach is impacted by selection bias, particularly as it relates to the number of clonal populations selected and quantitatively assessed from a given sample or condition. In addition, the specific ongoing changes in chromosome complements is expected to impact several key factors, including proliferation and viability, that direct population evolution and influence genetic drift over time. Finally, due to the need to assess clonally-derived populations over time, this approach is generally limited to experimental research/conditions and is not typically employed within the clinic due to the technical challenges associated with isolating individual cells and expanding them in vitro (e.g., artificial growth conditions) that may inadvertently introduce selective pressures that impact the outcomes.

Based on the limitations associated with the temporal approach, substantial efforts have been directed towards the development of single cell, endpoint approaches that are capable of quantifying the product of CIN, specifically the cell-to-cell heterogeneity contained within a given population rather than a specific cell lineage. A large number of single cell approaches including microscopy, flow cytometry, next generation DNA sequencing, and copy number analysis have been devised that are capable of assessing CIN phenotypes, such as changes in chromosome numbers by standard cytogenetic staining [23] or the use of chromosome enumeration probes and whole chromosome paints [24,25], along with more recent technological advancements including single cell DNA sequencing or copy number variation [26,27]. Single cell approaches have also been developed to quantify and compare surrogate markers of CIN (e.g., CIN-associated phenotypes), including micronucleus formation [28,29] and changes in nuclear areas [29] or human artificial chromosomes [30]. Conceptually, micronuclei are extra nuclear bodies that are found outside the primary nucleus and are hallmarks of CIN that typically arise due to chromosome missegregation events [31,32,33], while changes in nuclear areas and human artificial chromosomes are associated with small and large (i.e., ploidy) scale changes in DNA content, respectively [30,34,35,36,37,38]. These approaches typically involve quantitative imaging microscopy or flow cytometry that are each capable of rapidly assessing CIN-associated phenotypes in hundreds-to-thousands of cells [28,29,39]. In any case, numerous complementary single cell approaches have been developed that can provide critical insight into the prevalence of cell-to-cell heterogeneity and CIN within experimental and clinical contexts.

The fundamental goals of endpoint analyses are to quantify and statistically report on the cell-to-cell heterogeneity contained within and between experimental or clinical conditions while not employing population averaging, as that would mask the heterogeneity. The key benefits of these approaches are that they are rapid, cost effective, and amenable to screens involving experimental or clinical samples. However, unlike the temporal approaches, endpoint analyses provide only a single ‘snapshot’ of the genome and therefore provide only limited temporal insight into clonal or population evolution. In any case, endpoint approaches offer unparalleled insight into the level of cell-to-cell heterogeneity and population diversity associated with CIN. For example, these endpoint approaches and subsequent cytogenetic validation have been instrumental in expanding our understanding of the molecular determinants of CIN, which includes genes regulating chromosome cohesion and condensation [35,36,40,41,42], histone modifications [43,44,45,46], microtubule motor proteins [34,47], and ubiquitin regulating complexes [37,48,49]. Only once these single cell approaches are more readily applied in both experimental and clinical contexts will we begin to expand our current understanding of the intimate and causal relationships existing between CIN and cancer so that we can ultimately realize its clinical potential in enhancing case management and predicting clinical outcomes. What follows are brief discussions detailing our current understanding of CIN and its impact on: (1) cancer development and progression, (2) metastatic potential, (3) cancer prognosis, and (4) the development of precision medicine strategies to combat cancer.

## 3. The impact of CIN on Cancer Development and Progression

**Figure 1:**
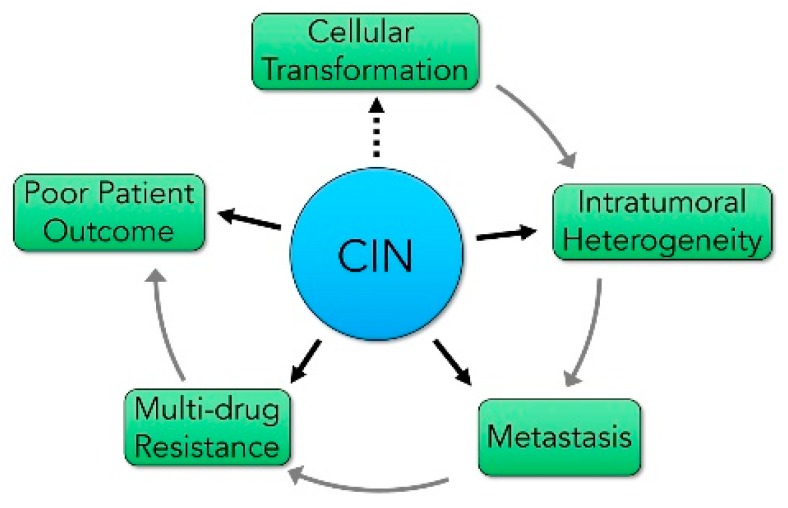
The impact of chromosome instability (CIN) on key features of cancer development, progression, and outcomes. A schematic depicting the central impact CIN has on early tumorigenic events (cellular transformation), tumor evolution (intratumoral heterogeneity), disease progression (metastasis), and the development of chemoresistance (multi-drug resistance), all of which are often associated with poor patient outcomes. Dotted lines identify proposed relationships, while solid lines identify established relationships.

Decades of fundamental and clinical research have shown that CIN has tremendous implications in cancer development, progression, and clinical outcomes (Figure 1). For example, CIN is associated with cellular transformation [9,50], tumor evolution and progression including intertumoral and intratumoral heterogeneity [51,52,53], metastasis [54,55,56], and the acquisition of drug resistance [57,58]. Thus, it is perhaps unsurprising that the presence of CIN is typically associated with worse patient outcomes [59,60,61,62]. However, it is interesting to note that there are a small subset of cancers in which the presence of CIN corresponds with improved survival [63,64,65]. Collectively, an extensive body of evidence exists to suggest that low-to-intermediate levels of CIN may be a driving force in cancer, while reduced tumor cell viability may be associated with extreme levels of CIN, which is a common therapeutic strategy employed to selectively kill cancer cells (reviewed in [66,67]). Nevertheless, and despite these associations, the aberrant molecular determinants inducing CIN remain poorly understood. In fact, of the ~2300 CIN genes (i.e., genes whose aberrant expression induces CIN) predicted to exist [34,68], fewer than 150 have been identified and validated to date. Furthermore, of those that have been identified, most encode functions within intuitive pathways that orchestrate chromosome dynamics and/or DNA repair, including chromosome segregation [12,69], sister chromatid cohesion [36,40,41], chromosome condensation [35,42], mitotic spindle dynamics [69,70,71], spindle assembly/mitotic checkpoint [72,73,74,75], kinetochore–microtubule attachments [34,76,77,78,79], centrosome dynamics [80,81,82], telomere biology [83,84,85], and DNA replication and repair [12,86,87]. Thus, significant efforts are required to greatly advance our limited understanding of the molecular determinants of CIN and their implications for cancer development, particularly as CIN pertains to intertumoral and intratumoral heterogeneity.

### The Relationship between CIN and Intertumoral and Intratumoral Heterogeneity

The genetic heterogeneity and diversity contained within tumors that are driven by CIN greatly impacts disease response and clinical outcomes and therefore pose significant challenges for clinical management [88]. Pathologists have long since recognized the morphologic changes and cell-to-cell heterogeneity present within tumors. In fact, the intertumoral heterogeneity observed between patients and the intratumoral heterogeneity present within the same patient form the basis for the histological classification of tumors [89,90]. In addition, oncologists have noted for decades the heterogeneous responses of tumors to chemotherapy—some tumors decrease in size (cytotoxic effect), whereas others remain unchanged (cytostatic) or continue to grow (refractory) [91,92]. While there are a number of biological explanations for these diverse responses, a unifying feature in the response may be the presence, extent, and level of CIN contained within a given tumor.

By its very nature, CIN drives intratumoral heterogeneity and has tremendous clinical implications for disease progression, response, and outcomes [53,88,93,94]. Thus, malignant tumors exhibiting CIN are highly heterogeneous at numerous levels, including those of the molecular (genetic), cellular, tissue, and human population [51,95]. For example, solid tumors are comprised of neoplastic cells constituting the tumor parenchyma and reactive stroma, as well as the structural component comprised of connective tissues, the extracellular matrix, blood vessels, and cells of the adaptive and innate immune systems [96]. Analyses of biopsied materials collected from distinct tumor regions [97,98] along with repeated sampling over time [22,99] have identified significant cell-to-cell heterogeneity and ongoing genetic changes, respectively. Furthermore, patient-specific sequencing has also identified genetic heterogeneity as existing between primary and metastatic lesions (see Section 4) within the same patient and that is indicative of CIN and tumor evolution [100,101]. Finally, while it is well established that individuals diagnosed with the same cancer type share key aberrant genetic events, most exhibit distinct genetic differences between patients (i.e., intertumoral heterogeneity) [102,103]. Collectively, the above observations are indicative of CIN, and thus it is not surprising that CIN plays a central role in driving the cell-to-cell heterogeneity that promotes disease development and progression, in addition to influencing patient outcomes.

## 4. CIN Influences the Metastatic Potential of Many Cancer Types

Metastasis is associated with advance stage disease and has a significant impact on morbidity and mortality rates [104], as ~90% of all cancer-associated deaths are due to the metastasis rather than the primary tumor [105]. Metastatic spread is also central to all tumor staging systems and is one of the most important determinants and negative predictors of clinical outcome [89,106]. Depending on the cancer type, metastases are frequently present in patients when they are first diagnosed with cancer [107]. For example, ~50% of colorectal cancers are diagnosed at stage III or IV [62], while up to 92% of high-grade serous ovarian cancers are newly diagnosed with late stage disease (III or IV) [108]. Metastatic lesions frequently share pathogenic driver events with the primary tumor, but they continue to evolve and develop distinct genetic alterations that promote further cell-to-cell, intra-tumoral, and inter-tumoral heterogeneity [109]. For example, metastatic cells must be genetically programmed to transition between cellular states (epithelial-to-mesenchymal or mesenchymal-to-epithelial transition) to adapt to distinct and variable growth conditions including both their routes of dissemination (hematogenous versus lymphatic versus direct seeding) and their final metastatic environment (tissue or organ) [110]. Thus, identifying the molecular determinants driving metastatic spread is critical to optimize the clinical management of the disease.

Recent genetic studies have focused on the origins and evolution of metastatic disease, which has led to the development of two evolutionary metastatic models [111,112]—linear and parallel progression—that describe the potential processes driving metastatic spread, with a particular focus on the clonal relationship between the primary tumor and its metastatic deposits (see [101]). Briefly, the linear progression model stipulates that cells in the primary tumor undergo a series of genetic alterations, such that metastatic clones are seeded late in the course of tumor evolution. Thus, there is minimal genetic divergence between cells in the primary tumor and the metastatic lesions [113,114]. Alternatively, the parallel progression model states that a metastatic clone is derived early during disease development, and, so there is a large degree of genetic divergence between cells in the primary and metastatic sites.

The genesis of metastatic disease is a complex phenomenon, involving the coordinated expression and regulation of multiple genes involved in multiple pathways at both the primary and metastatic sites [115]. In both metastatic models, CIN may be the driving force behind the extensive genetic changes that ultimately produce the initiating metastatic clone. CIN may enable cells to readily adapt and evolve, such that they undergo the relevant genetic changes required for metastasis to occur [18,94]. For example, the loss of *CDH1* (E-cadherin) expression, a cell-to-cell adhesion molecule, is a key driver of epithelial-to-mesenchymal transition, a pathogenic event associated with enhanced invasive and metastatic potential [116,117]. The epithelial-to-mesenchymal transition is a critical change in which epithelial cells lose their polarity and transition into a more mesenchymal-like state. This transition enables cells to become increasingly motile and to develop the cellular apparatus required to invade the basement membrane, the passage through the extracellular matrix and intravasate into blood vessels during the metastatic process [110], which may be driven, at least in part, by CIN. For example, Bakhoum and colleagues [54] recently demonstrated in mouse models that CIN promotes metastasis through a cytosolic DNA response. More specifically, they showed that chromosome segregation errors lead to the formation of micronuclei (see Section 2.2) that can rupture and spill their genomic DNA into the cytosol, which in turn leads to the activation of the cGAS-STING (cyclin GMP–AMP synthase-stimulator of interferon genes) cytosolic DNA-sensing pathway and downstream non-canonical NF-κB signaling that promotes the expression of inflammation and epithelial-to-mesenchymal transition genes required for metastasis to occur. Importantly, they showed that suppression of CIN markedly delayed metastasis, whereas ongoing segregation errors (e.g., CIN), promoted cellular invasion and metastasis in a STING-dependent manner, thus establishing a causal relationship between CIN and metastasis. Nevertheless, additional research is required to fully elucidate the spatio-temporal relationship and impact of CIN on the metastatic process.

## 5. CIN and Cancer Prognosis

The presence of CIN is most often associated with poor patient outcomes in numerous cancer types, including breast, cervical, colon, endometrial, gastric, head and neck, lung, ovarian, and hematologic cancers [118]. This negative association has been proposed to primarily arise from the intratumoral heterogeneity induced by CIN, which enables a sub-populations of cells within a tumor to acquire more aggressive and invasive phenotypes that drive disease progression, metastasis, and drug resistance. CIN is observed in up to 85% of all sporadic colorectal cancer [119], where it is associated with poor prognosis and is an independent prognostic marker. For example, stage IV colorectal cancers generally have a higher level of CIN relative to stage I, although there is no stepwise and increasing progression pattern across all four stages [62,120]. Higher levels of CIN are also observed in metastatic lesions, relative to non-metastatic colorectal cancers [62]. Collectively, these findings suggest that high levels of CIN may confer more aggressive and invasive cellular phenotypes that correlate with an increased metastatic potential.

The presence of CIN has also been used to identify both chemoresistance and drug sensitivity to specific anticancer drugs [121,122,123] and may ultimately enable the custom tailoring of specific chemotherapeutic regimens to a given patient’s tumor. Beyond colorectal cancer, high levels of CIN are also associated with intrinsic drug resistance in many cancer types [57,88]. For example, Spears et al. [124] showed that the presence of CIN (as assessed by a four gene signature) predicts patients who will benefit from anthracyclines (doxorubicin) treatments in breast cancer, while Swanton and colleagues [123] showed that ovarian cancers with high levels of CIN exhibit intrinsic resistance to taxanes (paclitaxel) but retain platinum-based sensitivity (carboplatin). Accordingly, these data suggest that CIN, or more likely, the level of CIN, may confer sensitivity or resistance to specific anti-neoplastic drugs. Researchers have also shown that CIN can predict which patients are most likely to benefit from specific treatments, such as bevacizumab (an anti-angiogenic monoclonal antibody targeting vascular endothelial growth factor A [VEGF-A]), a key drug used in the treatment of colorectal and lung cancers [125]. Based on these collective observations, the authors suggest that the presence and level of CIN may be a useful tool that could assist oncologists in stratifying patient cohorts to distinguish those who will benefit from a given treatment from those who will not.

Beyond its implications in therapeutic responses, the presence of CIN has also been used to predict metastasis [126,127]. Accordingly, determining the presence and level of CIN within a given patient sample may be useful to predict the risk of metastatic disease, chemo-resistance, and overall patient survival [64,128]. In 2006, Carter and colleagues [118] developed a computational method to characterize CIN in breast cancers that is based on the concurrent expression of established CIN genes. Conceptually, this method assesses either a 25 (CIN25) or 70 (CIN70) gene signature based on the altered expression of the top 25 or 70 genes, respectively. In agreement with the above findings, higher levels of CIN were observed within the metastatic lesions relative to the primary tumor site. Interestingly, the authors were able to stratify grade 1 and grade 2 tumors based on their CIN25 gene signatures, and they further determined that high CIN25 tumors were generally associated with worse clinical outcomes. This seminal work suggested that in some cancers, CIN may be a stronger predictor of clinical outcome than conventional prognosis determinants such as tumor grade and stage. Indeed, subsequent work has shown that the aberrant expression of the CIN70 gene signature is predictive of poor outcome in many cancer types, including cervical carcinoma, lymphoma, lung adenocarcinoma, glioma, medulloblastoma, and mesothelioma, and it is predictive of metastatic spread in primary, untreated, gastrointestinal stromal tumors [63,118,129]. Similarly, DNA ploidy, which may be reflective of CIN, has been used as a prognostic marker in multiple cancer types, including lung squamous cell carcinoma, pancreatic adenocarcinoma, ovarian epithelial carcinomas, gastric adenocarcinoma, endometrial carcinoma, prostatic adenocarcinoma, pediatric neuroblastoma, and rhabdomyosarcoma [130,131,132,133,134,135,136]. Often, this has been found to be the most important prognostic marker, independent of primary tumor site, histologic type, or TNM (tumor, node, metastasis) staging status. Collectively, the above observations suggest that the presence of CIN may provide insight into drug sensitivities and patient outcomes.

Paradoxically however, high levels of CIN also correlate with improved clinical outcomes and survival, although this has only been observed in specific cancer types, including a subset of breast, ovarian, lung, and gastric cancers [63,64]. While the mechanisms accounting for these contradictory findings remain unclear, it has been suggested that high or extreme levels of CIN may be less compatible with viability than low levels of CIN. Thus, tumor cells with extreme levels of CIN likely die and are lost from the population (reviewed in [137]), whereas those with low levels of CIN may be more aggressive and promote tumor development, progression, metastasis, and drug resistance. Collectively, the above data suggest that it is more likely the level of CIN (i.e., low versus high), rather than the tumor type itself, that discerns whether CIN is associated with better or worse clinical outcomes. This possibility is further underscored by the many therapeutic strategies (currently employed or under development) that now seek to induce extreme levels of CIN to enhance the killing of cancer cells [66,67,138,139,140,141,142,143]. Accordingly, it is becoming increasingly important to determine the extent and level of CIN within tumors, whether primary or metastatic, as this critical information may hold tremendous diagnostic, prognostic, and therapeutic value.

## 6. CIN and its Impact on Precision Medicine Strategies

**Figure 2:**
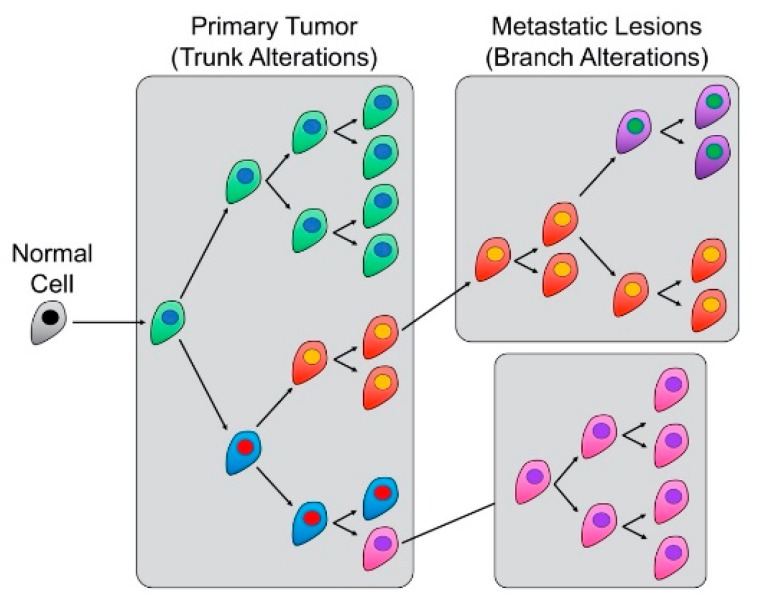
CIN drives trunk and branch alterations to contribute to tumor evolution and metastasis. Illustration showing the tree-like ‘trunk’ and ‘branch’ alterations driven by CIN. In general, trunk alterations are early events that are conserved in all subsequent cellular progeny, whereas branch alterations are subsequent genetic alterations that direct clonal evolution and intratumoral heterogeneity to drive disease progression, metastasis, and drug resistance. Note that the color changes coincide with cells that have accrued additional genetic alterations (e.g., CIN).

The preceding sections highlight the impact CIN has on inter- and intra-tumoral heterogeneity, which have significant influence over the spatio-temporal development and evolution of malignant tumors [18,94]. Frequent drivers of cell-to-cell heterogeneity often include extrinsic factors such as pH, hypoxia, paracrine signaling, stromal interactions, and drugs [144,145], which can potentiate or even induce CIN by themselves. For example, many of these factors increase cell-to-cell heterogeneity by modulating intracellular signaling pathways or by exerting selective pressures that influence cellular proliferation and/or viability; therefore, these factors can direct clonal evolution [146]. In this regard, numerous research teams have begun to map and study the specific pathogenic events and their temporal order of appearance by creating ancestral ‘trees’ to describe a tumor’s evolution, in much the same manner that classical phylogenetic trees describe species evolution [147]. In keeping with the ‘tree’ analogy (Figure 2), early pathogenic events are commonly referred to as ‘trunk’ or truncal alterations, whilst late occurring alterations (e.g., driving metastatic changes) are referred to as ‘branch’ alterations (see also [148]), both of which have implications for treatment strategies and outcomes.

A central goal of many researchers and oncologists is to identify and distinguish between truncal and branch alterations to glean context-specific insight into disease etiology, as this detailed information is highly relevant in clinical management. For example, distinguishing between truncal and branch alterations (Figure 2) would identify key molecular events driving early (e.g., cellular transformation and disease development) and late (e.g., disease progression, metastasis, and drug resistance) disease events, which is critical when developing precision medicine strategies aimed at exploiting those molecular defects (reviewed in [67]). For example, as truncal alterations occur early and are present in all cellular progeny, emerging strategies are being devised that selectively target those truncal events to provide maximal therapeutic impact [149]. Interestingly however, branch alterations are also being investigated for their clinical utility in therapeutic targeting. Recall that branch alterations occur late in disease and are associated with disease progression and metastatic disease, but they are also considered potential sources of therapeutic resistance [145]. Consequently, therapeutically exploiting branch alterations is predicted to limit or prevent chemoresistance and disease recurrence. Accordingly, many research teams are now investigating combinatorial approaches, leveraging both trunk and branch alterations to achieve maximal therapeutic benefit and to dramatically improve health outcomes for those living with cancer [150]. Alternatively, Li and colleagues [151] proposed another strategy, referred to as the “Evolutionary Trap,” that seeks to target both karyotypic diversity and fitness. In principle, the goal is to selectively condition or channel a karyotypically diverse population into a dominant population with a predictable sensitivity to a particular drug. Though only experimentally demonstrated in budding yeast, this approach may one day prove useful in a human context.

### 6.1. The Impact of CIN on Therapeutic Targeting

**Figure 3:**
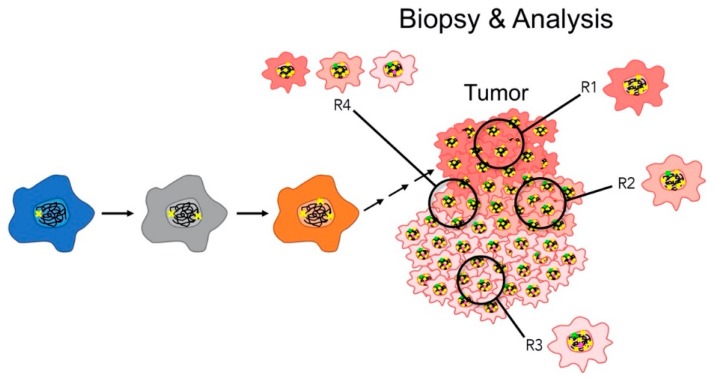
Impact of CIN on regional biopsies and subsequent analyses. A schematic showing how CIN (represented by arrows) induces genetic diversity (colored ‘x’) within a given tumor (represented by color changes). Note that the ability to glean tumor-specific insight (trunk versus branch alterations) into the aberrant genetics driving tumor development and progression is impacted by regional sampling and the composition and clonality of the tumor cells contained within the biopsied region. Single region bias is demonstrated by the four distinct regions (R1–4), which exhibit variation in both the type of clones identified (R1–3) and in the composition (presence and frequency) of the clones (R4) contained within a given biopsy.

Developing therapeutic strategies that simultaneously target trunk and branch alterations is a relatively simple concept; however, CIN adds an additional layer of complexity that impacts the ability to accurately identify the exploitable genetic alterations. It is generally accepted that CIN is an early etiological event in the development of numerous cancer types, as it is detected in dysplastic/precancerous lesions [108,152,153,154] and can induce cellular transformation [9,50]. Thus, CIN genes, or those genes whose aberrant expression induces CIN, are frequently viewed as trunk alterations [51,94]. Unfortunately, the ongoing cell-to-cell heterogeneity that is induced by defects in CIN genes renders it challenging to identify additional trunk alterations and extremely challenging to identify downstream branch alterations. This layer of complexity is further compounded when only single region biopsies are collected and assessed from a given patient, as single region sampling is unlikely to reflect the level and extent of genetic heterogeneity comprising an entire tumor (Figure 3). Moreover, since CIN drives tumor progression and evolution (i.e., intratumoral heterogeneity), the genetic profile of a single biopsy does not provide the spatio-temporal resolution required to appropriately monitor the disease response to a given anti-neoplastic, including the development of drug-resistant clones and, ultimately, chemoresistance. This is especially relevant, as a protracted disease course inevitably leads to extensive genetic diversity within a CIN tumor cell population, such that the cells present in a late stage tumor are genetically disparate from those biopsied or removed at the time of primary surgery in early stage disease [155]. Furthermore, as CIN confers multidrug resistance [57,58], single agent strategies typically fail due to the ineffectiveness of the treatment across the entire tumor population and/or the adaptive nature of the cells exhibiting CIN. Thus, the accuracy of prognostic stratification will likely provide limited insight, as the diagnostic information is restricted to the region that is analyzed. This regional bias could conceivably be partially overcome with multi-region sampling (Figure 3), but the true extent of cell-to-cell genetic heterogeneity may still remain underestimated, as low frequency variants are not easily detected when using conventional genomic analyses [156].

As indicated above, CIN is a driver of genetic and intratumoral heterogeneity that exerts selection pressures on cancer evolution to direct disease progression, metastasis, chemoresistance, and disease recurrence [88,157]. Thus, intimate knowledge and the evaluation of intratumoral heterogeneity within primary tumors are key factors required to improve patient outcomes. Moreover, the cell-to-cell heterogeneity imparted by CIN yields an even greater challenge in metastatic disease, as the metastatic deposits accrue additional branch alterations that further distinguish them from the primary tumor. Accordingly, the multi-region profiling of both primary and metastatic regions (or CTCs) is required to overcome the complex and differing genetic landscapes to ultimately identify actionable targets for maximum therapeutic response. Unfortunately however, multi-region and repeat tissue sampling is not without its own caveats—it is logistically challenging, costly, and labor intensive. Perhaps even more important is that multi-region and repeat sampling are associated with increased risks for metastatic spread/seeding due to the disruption of the primary or metastatic tumor architecture [20,21]; hence, it is not routinely employed within the clinic.

There are currently a number of emerging and alternative approaches that seek to eliminate the risks associated with multi-region/repeat samples, and these include liquid biopsies and blood or body fluid-based collections [158,159]. These minimally invasive approaches enable the assessment of CTCs, potentially providing simultaneous insight into both primary and metastatic sites. Beyond the genetic assessments (e.g., trunk versus branch alterations) enabled through these approaches, the presence of CTCs in both early and late metastatic stage disease corresponds with worse patient outcomes and decreased survival rates [158,159]. Even more relevant to therapeutic targeting, decreases in CTCs following treatment are associated with a better overall survival, and, thus, quantitative changes in CTCs are now being employed as markers of early treatment response [160]. Perhaps most importantly, various genetic assessments, including single cell DNA sequencing, copy number alterations, and the myriad of CIN-based analyses may provide additional and critical clinical information. For example, CTCs isolated from liquid biopsies are more readily amenable to sequential sampling than traditional tumor biopsies, and they can provide ‘real-time’ insight into tumor genetics, CIN, and intratumoral heterogeneity that may prove useful in monitoring disease progression, treatment responses [160], and/or modifying treatment decisions [147].

## 7. Conclusions

There is a resurgence and increasing research focus on the impact CIN has on cancer development and clinical outcomes. CIN drives cell-to-cell heterogeneity and has profound effects on the cancer cell genome, tumor pathogenesis, tumor evolution, metastatic spread, and treatment options and success [94,157]. CIN is an important mechanism by which cancer cells acquire extensive genetic alterations that ultimately influence and direct tumor behavior and evolution. Thus, efforts aimed at identifying and characterizing the molecular determinants of CIN will provide critical insight into disease biology that will be essential to enhance patient risk stratification and maximize therapeutic response and clinical outcomes. Recent technological advances have allowed for a deeper understanding of tumor genomes, with the repeated identification of the pervasive nature and prevalence of CIN in numerous cancer types [4]. Nevertheless, a significant obstacle in this endeavor is our limited understanding of the causative mechanisms driving CIN, as well as the technical challenges associated with measuring the spatio-temporal aspects of CIN in clinical settings. Accordingly, as technologies continue to advance and become more affordable, greater insight into CIN and its impact in cancer will be gleaned that will enable the development of novel therapeutic strategies aimed at improving the lives and outcomes of those living with cancer.
